# Region-specific dendritic simplification induced by Aβ, mediated by tau via dysregulation of microtubule dynamics: a mechanistic distinct event from other neurodegenerative processes

**DOI:** 10.1186/s13024-015-0049-0

**Published:** 2015-11-05

**Authors:** Nataliya Golovyashkina, Lorène Penazzi, Carlo Ballatore, Amos B. Smith, Lidia Bakota, Roland Brandt

**Affiliations:** Department of Neurobiology, University of Osnabrück, Barbarastrasse 11, 49076 Osnabrück, Germany; Department of Chemistry, School of Arts and Sciences, University of Pennsylvania, Philadelphia, PA 19014 USA; Center for Neurodegenerative Disease Research, Department of Pathology and Laboratory Medicine, Perelman School of Medicine, University of Pennsylvania, Philadelphia, PA 19104 USA

**Keywords:** Dendritic simplification, Microtubules, Alzheimer’s disease, Tau protein, Amyloid beta, Epothilone D

## Abstract

**Background:**

Dendritic simplification, a key feature of the neurodegenerative triad of Alzheimer’s disease (AD) in addition to spine changes and neuron loss, occurs in a region-specific manner. However, it is unknown how changes in dendritic complexity are mediated and how they relate to spine changes and neuron loss.

**Results:**

To investigate the mechanisms of dendritic simplification in an authentic CNS environment we employed an ex vivo model, based on targeted expression of enhanced green fluorescent protein (EGFP)-tagged constructs in organotypic hippocampal slices of mice. Algorithm-based 3D reconstruction of whole neuron morphology in different hippocampal regions was performed on slices from APP_SDL_-transgenic and control animals. We demonstrate that induction of dendritic simplification requires the combined action of amyloid beta (Aβ) and human tau. Simplification is restricted to principal neurons of the CA1 region, recapitulating the region specificity in AD patients, and occurs at sites of Schaffer collateral input. We report that γ-secretase inhibition and treatment with the NMDA-receptor antagonist, CPP, counteract dendritic simplification. The microtubule-stabilizing drug epothilone D (EpoD) induces simplification in control cultures *per se*. Similar morphological changes were induced by a phosphoblocking tau construct, which also increases microtubule stability. In fact, low nanomolar concentrations of naturally secreted Aβ decreased phosphorylation at S262 in a cellular model, a site which is known to directly modulate tau-microtubule interactions.

**Conclusions:**

The data provide evidence that dendritic simplification is mechanistically distinct from other neurodegenerative events and involves microtubule stabilization by dendritic tau, which becomes dephosphorylated at certain sites. They imply that treatments leading to an overall decrease of tau phosphorylation might have a negative impact on neuronal connectivity.

## Background

Alzheimer’s disease (AD) is characterized by the neurodegenerative triad of spine changes, dendritic simplification and neuron loss [1]. The pathologic changes are accompanied by the formation of extracellular plaques, composed of Aβ peptides, and intracellular neurofibrillary tangles containing tau in a hyperphosphorylated state [2, 3]. While the development of spine changes and neuron loss has been addressed in various animal and culture models [4–7], much less is known about how changes in dendritic complexity are mediated. Spires et al. [7] reported dendritic changes in plaque developing mice and Wu et al. [8] provided evidence that activation of the calcium-dependent phosphatase calcineurin is involved in Aβ-induced dendritic simplification, however it remains unclear how these changes are mediated. The lack of knowledge is noteworthy since dendritic simplification, which has long been recognized in AD, is likely to contribute to brain malfunction to a major extent and is considered to be a forerunner to neuronal loss [9].

A number of careful morphological studies of human brain material have established that alterations in dendritic arbor are regionally specific in AD [10]. In the hippocampus, dendritic extent is largely reduced in CA1 pyramidal neurons [11], while no changes were observed in CA3 neurons [12, 13]. The differences appear not to be due to postsynaptic dendritic regression since no excess loss of CA3 pyramidal neurons was observed [14]. Loss of spines is more widespread [15], suggesting that dendritic simplification develops independent from spine changes and neuronal loss. How changes in neuronal morphology develop during disease is however largely unknown.

Neuronal morphology is highly dependent on the organization of the cytoskeleton with microtubules playing an important role in regulating dendritic arborization [16]. The microtubule-associated tau proteins, known to modulate microtubule dynamics in neurons [17], are present and functionally active to a major extent in dendrites [18, 19] and may also be involved in dendritic remodeling during health and disease [20]. However, whether Aβ and tau are involved in dendritic simplification and whether they functionally interact to execute these changes is unknown.

To scrutinize the mechanisms of dendritic simplification in an authentic CNS environment, we employed a previously established ex vivo model based on targeted expression of EGFP-tagged constructs in organotypic hippocampal slices [5]. We demonstrate that dendritic simplification is restricted to principal neurons of the CA1 region, and is induced by Aβ, mediated by tau through NMDA receptor activation. We provide evidence that dendritic simplification involves dysregulation of microtubule dynamics by dendritic tau, which becomes dephosphorylated at certain sites, and is mechanistically distinct from spine changes and neuron loss.

## Results

### Induction of dendritic simplification requires the action of human tau on an APP_SDL_ transgenic background

**Fig. 1:**
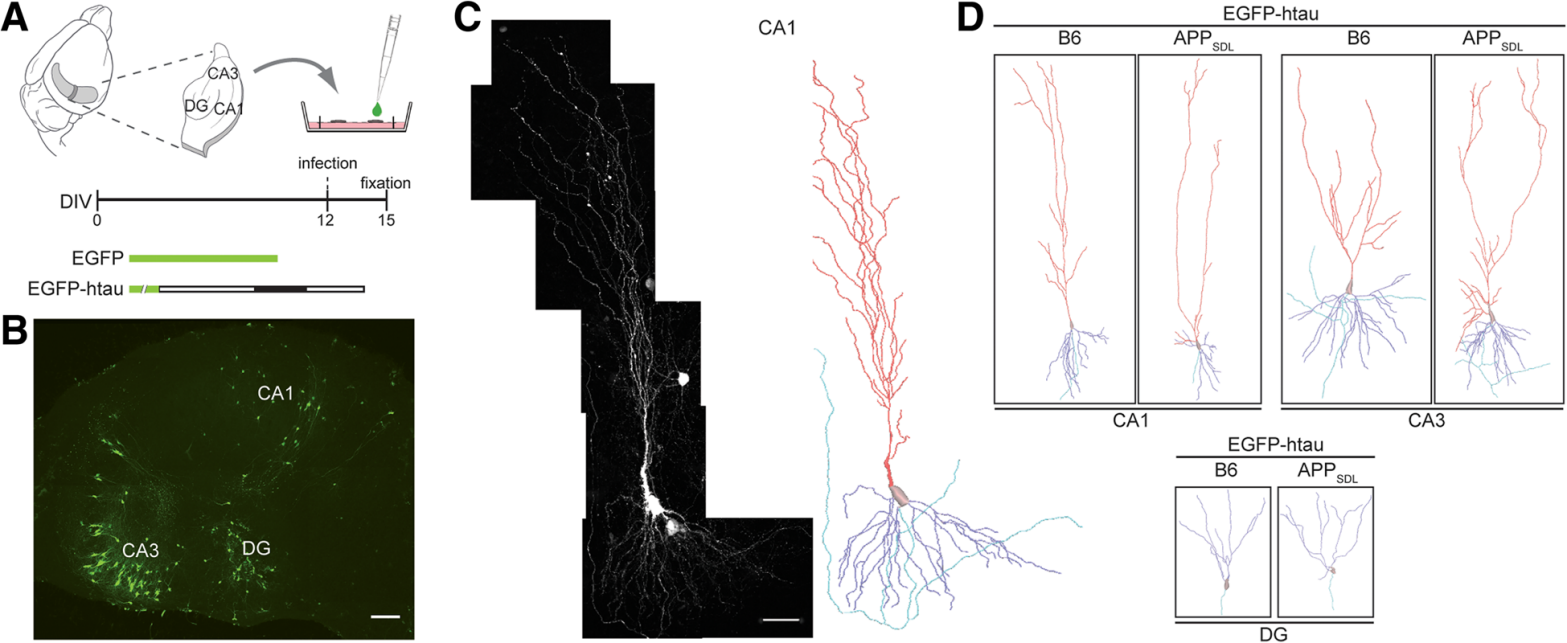
Analysis of dendritic simplification in an ex vivo model of Alzheimer’s disease. **a** Outline of the experimental approach. 400 μm-thick hippocampal sections from APP_SDL_ transgenic or non-transgenic mice were cultured using the membrane interface technique. Sindbis virus infection to express EGFP-tagged constructs was performed at day 12 in vitro (DIV). Slices were fixed three days later as indicated on the time line. **b** Fluorescence micrograph of a whole slice showing infected and EGFP-expressing neurons in DG, CA1 and CA3 regions. Scale bar, 150 μm. **c** High resolution fluorescence micrograph of a CA1 pyramidal neuron after semi-automated stitching (*left*) and 3D reconstruction (*right*). The apical part of the dendritic tree is indicated in red, the basal part in blue and the axon in turquoise. Scale bar, 50 μm. **d** 3D reconstructions of representative CA1, CA3 and DG neurons from APP_SDL_ transgenic and non-transgenic (B6) mice after expression of EGFP-htau

We employed an ex vivo model of AD using organotypic hippocampal slice cultures from APP_SDL_ transgenic mice in combination with Sindbis virus-mediated expression of EGFP-tagged constructs. We demonstrated previously that this approach permits analysis of cell death and synaptic changes with respect to the functional interaction between Aβ and tau pathology [5]; here we have extended the approach to determine region-specific changes in dendritic morphology. Virus infections were performed at 12 days in vitro (DIV) and slices were analyzed 3 days later (Fig. 1a). At these conditions efficient neuronal expression of EGFP with low toxicity was obtained [21]. We have also shown previously that the expression levels of different EGFP-tau constructs are similar [5, 21]. Virus titer was adjusted to achieve low infection rate in CA1, CA3 and DG, which permitted 3D imaging of single neurons by high-resolution cLSM with minimal overlap of dendritic arbors (Fig. 1b). Image tile stacks were stitched into single neurons and the morphology of whole neurons was reconstructed in 3D by automated digitalization (Fig. 1c) [22]. The detection gain was adjusted to capture the full arbor, including smaller secondary and tertiary dendrites, irrespective of the EGFP-construct used. Comparing neuronal morphology after virus infection in slices prepared from APP_SDL_ transgenic mice versus B6 controls permitted determination of the effect of the APP transgene on dendritic morphology in the apical (red) and basal (blue) arbor in principal neurons from the different hippocampal regions (Fig. 1d).

**Fig. 2:**
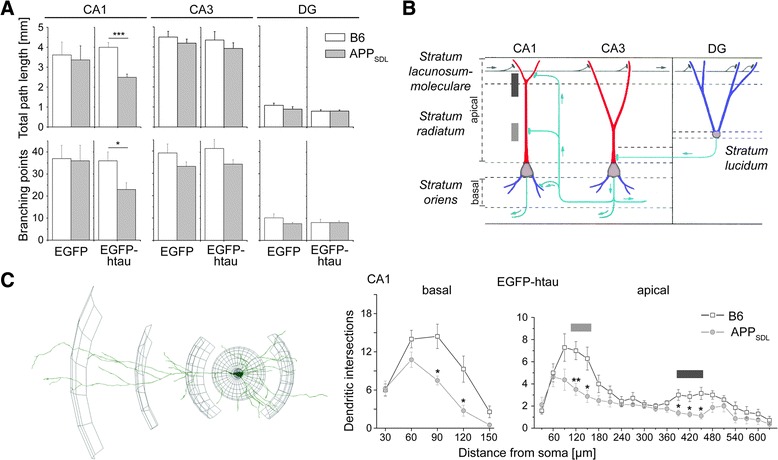
Effect of tau and APP on dendritic morphology of principal hippocampal neurons. **a** Quantitative analysis of dendritic complexity as determined from total path length (*top*) and number of branching points (*bottom*). Expression of EGFP-htau induces dendritic simplification in CA1 neurons from APP_SDL_ transgenic mice. **b** Schematic representation showing the major connectivity in different layers of the hippocampus. Gray boxes indicate sites of Schaffer collateral input in apical dendrites of CA1 neurons. **c** 3D Sholl analysis of the basal and apical dendritic tree in CA1 neurons. A schematic representation illustrating the procedure to determine dendritic intersections is shown left. Significant changes occur at two segments in the apical dendrite, which are indicated by gray boxes corresponding to the Schaffer collateral input as shown in (**b**). Statistical evaluation was performed using two-tailed, unpaired Student’s *t* test for comparison of the two genotypes in (**a**) and (**c**). Values are shown as mean and s.e.m. *, *p* < 0.05, **, *p* < 0.01, ***, *p* < 0.001. See Table 1 for details on the statistics

For a quantitative assessment of potential changes in dendritic arborization, we determined the total path length and the number of branching points in principal neurons from CA1, CA3 and DG. Expression of EGFP alone on APP_SDL_ transgenic background induced only slight dendritic simplification, which did not reach significance. In contrast, we observed that EGFP-htau expression on APP_SDL_ transgenic background induced pronounced and significant dendritic simplification in CA1 neurons as indicated by a reduction of total path length and number of branching points by 38 % and 30 %, respectively (Fig. 2a, left). No change was seen in CA3 pyramidal neurons and granule cells of the DG (Fig. 2a, middle and right). Human tau expression on non-transgenic background did not induce morphological changes, indicating that dendritic simplification in the CA1 region requires the combination of human tau and APP_SDL_ transgenic background.

### Dendritic simplification occurs at sites of Schaffer collateral input in CA1 neurons

CA1 pyramidal neurons receive different inputs in specific subregions of the dendritic tree (Fig. 2b). To determine subregional differences in the induction of dendritic simplification, 3D Sholl analysis [23] was performed on basal and apical branches to quantitate changes in the intersection frequency of dendrites as a function of the distance from the cell body (Fig. 2c, left). On the APP transgenic background we observed a reduction of dendritic complexity in the entire basal part of the tree (Fig. 2c, right). In contrast, in the apical part, dendritic simplification was induced specifically in two segments of the tree. Responsive segments were localized at ~15-25 % and ~60-70 % of total apical length and correlated with regions, where most input from Schaffer collaterals occurs (see Fig. 2b).

### γ-Secretase inhibition and treatment with NMDA receptor inhibitors counteract dendritic simplification

**Fig. 3:**
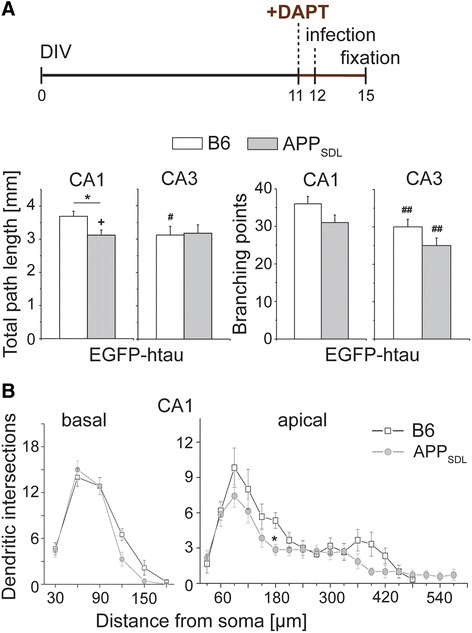
Effect of γ-secretase inhibition on dendritic simplification. **a** Total path length and number of branching points of CA1 and CA3 neurons in the presence of the γ-secretase inhibitor DAPT. Schematic representation of the time line of the respective experiment is shown on top. DAPT reduces simplification in CA1 neurons. **b** Sholl analysis of basal and apical parts of the dendritic tree in CA1 neurons after DAPT treatment. DAPT completely abolishes simplification of APP_SDL_ transgenic cultures in the basal part and reduces simplification in the apical part of the dendritic tree. (+) indicates significant increase, (#,##) significant decrease compared to the respective condition without the drug. Statistical evaluation was performed using one way ANOVA with post hoc Fisher’s LSD test for multiple comparisons (**a**) and Student’s *t* test for comparison of the two genotypes (**b**). Values are shown as mean and s.e.m. *(#)(+), *p* < 0.05, (##), *p* < 0.01. See Table 1 for details on the statistics

Dendritic simplification could be induced by the presence of mutated APP or might require the generation of Aβ through γ- and β-secretase mediated cleavage of APP_SDL_. To distinguish between these possibilities, we pretreated the culture with the non-transition state γ-secretase inhibitor DAPT in order to reduce Aβ generation [24]. Treatment with DAPT was started one day before infection with EGFP-htau expressing virus (Fig. 3a, top). DAPT reduced the negative effect of tau and APP on dendritic complexity in CA1 neurons as judged by a significant increase in total path length compared to untreated cultures of the same genotype (Fig. 3a, bottom). Sholl analysis of CA1 neurons revealed that dendritic simplification was completely abolished in basal dendrites and reduced in apical segments (Fig. 3b). The data indicate that dendritic simplification is induced by Aβ rather than mutated APP.

**Fig. 4:**
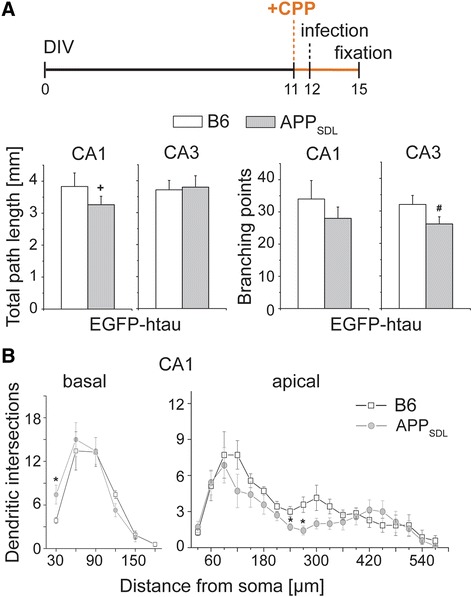
Effect of the NMDA receptor antagonist CPP on dendritic simplification. **a** Total path length and number of branching points of CA1 and CA3 neurons in the presence of the NMDAR antagonist CPP. Schematic representation of the time line of the respective experiment is shown on top. CPP reduces simplification in CA1 neurons. **b** Sholl analysis of basal and apical parts of the dendritic tree in CA1 neurons after CPP treatment. CPP completely abolishes simplification of APP_SDL_ transgenic cultures in the basal part and reduces simplification in the apical part of the dendritic tree. (+) indicates significant increase, (#) significant decrease compared to the respective condition without the drug. Statistical evaluation was performed using one way ANOVA with post hoc Fisher’s LSD test for multiple comparisons (**a**) and Student’s *t* test for comparison of the two genotypes (**b**). Values are shown as mean and s.e.m. *(#)(+), *p* < 0.05. See Table 1 for details on the statistics

It has previously been shown that Aβ acts via NMDA receptor (NMDAR) activation to induce spine changes and neuron loss [5, 25, 26]. To test whether NMDAR activation is also involved in mediating dendritic simplification, we added the competitive NMDAR antagonist CPP [27] to the cultures one day before infection with EGFP-htau expressing virus (Fig. 4a, top). CPP also abolished the negative effect of tau and Aβ on dendritic complexity in CA1 neurons similar to DAPT (Fig. 4a, bottom, and b), demonstrating that dendritic simplification is mediated by NMDAR activation. It should also be noted that both drugs induced some dendritic simplification in CA3 neurons, which might indicate sensitivity of the CA3 neurons to the drugs themselves.

**Table 1:** Dendritic simplification in an ex vivo model of Alzheimer’s disease. Summary representation of the effect of the expressed construct, the genotype of the mouse and pharmacological treatments on total path length (A) and number of branching points (B). Statistical evaluation was performed using two-tailed, unpaired Student’s *t* test for comparison of the two genotypes under control conditions (untreated), and one way ANOVA with post hoc Fisher’s LSD test for multiple comparisons of drug treatments or different tau constructs. *P* values, which represent statistical significance (*p*≤0.05) are indicated in bold, except when the alpha level of ANOVA was above 0.05

A. Total path length									
Construct	Condition	Region	Genotype	Mice (n)	Cells (n)	Mean ± s.e.m (based on cell number) in mm	Student’s test		
							*p* values (B6 vs. APP)		
EGFP	untreated	CA1	B6	5	9	3.113±0.465	*p* = 0.775		
EGFP	untreated	CA1	APP_SDL_	5	5	3.364±0.707			
EGFP	untreated	CA3	B6	7	8	4.490±0.292	*p* = 0.401		
EGFP	untreated	CA3	APP_SDL_	5	7	4.187±0.202			
EGFP	untreated	DG	B6	6	6	1.223±0.232	*p* = 0.275		
EGFP	untreated	DG	APP_SDL_	5	7	0.911±0.161			
EGFP-htau	untreated	CA1	B6	4	7	3.996±0.237	***p*** **= 0.0003**		
EGFP-htau	untreated	CA1	APP_SDL_	6	8	2.491±0.163			
EGFP-htau	untreated	CA3	B6	5	6	4.344±0.423	*p* = 0.428		
EGFP-htau	untreated	CA3	APP_SDL_	5	6	3.920±0.283			
EGFP-htau	untreated	DG	B6	5	10	0.813±0.071	*p* = 0.888		
EGFP-htau	untreated	DG	APP_SDL_	7	17	0.826±0.051			
Construct	Condition	Region	Genotype	Mice (n)	Cells (n)	Mean ± s.e.m (based on cell number) in mm	One way ANOVA (together with EGFP-htau untreated, both genotypes, same region)	*p* values (B6 vs. APP)	*p* values ( vs. untreated EGFP-htau same region and same genotype)
EGFP-htau	DAPT	CA1	B6	4	6	3.691±0.154	**F (3,24) = 14.05 ;** ***p*** **< 0.0001**	***p*** **= 0.0440**	*p* = 0.2657
EGFP-htau	DAPT	CA1	APP_SDL_	4	7	3.122±0.154			***p*** **= 0.0182**
EGFP-htau	DAPT	CA3	B6	5	6	3.122±0.258	**F (3,22) = 3.634 ;** ***p*** **= 0.0287**	*p* = 0.8981	***p*** **= 0.0133**
EGFP-htau	DAPT	CA3	APP_SDL_	4	8	3.177±0.249			*p* = 0.0940
EGFP-htau	CPP	CA1	B6	5	7	3.671±0.289	**F (3,25) = 7.781 ;** ***p*** **= 0.0008**	*p* = 0.2469	*p* = 0.3517
EGFP-htau	CPP	CA1	APP_SDL_	6	7	3.265±0.263			***p*** **= 0.0279**
EGFP-htau	CPP	CA3	B6	6	6	3.714±0.293	F (3,23) = 0.5765 ; *p* = 0.6363	*p* = 0.8658	*p* = 0.2483
EGFP-htau	CPP	CA3	APP_SDL_	7	9	3.797±0.357			*p* = 0.8023
EGFP-htau	EpoD	CA1	B6	7	8	3.038±0.288	**F (3,27) = 7.040 ;** ***p*** **= 0.0012**	*p* = 0.5142	***p*** **= 0.0092**
EGFP-htau	EpoD	CA1	APP_SDL_	8	8	2.820±0.241			*p* = 0.3273
EGFP-htau	EpoD	CA3	B6	7	8	3.603±0.186	**F (3,23) = 5.757 ;** ***p*** **= 0.0043**	***p*** **= 0.0416**	*p* = 0.0527
EGFP-htau	EpoD	CA3	APP_SDL_	7	7	2.853±0.126			***p*** **= 0.0089**
EGFP-Ala htau	untreated	CA1	B6	5	7	2.797±0.268	**F (3,24) = 8.839 ;** ***p*** **= 0.0004**	*p* = 0.1005	***p*** **= 0.0011**
EGFP-Ala htau	untreated	CA1	APP_SDL_	4	6	3.370±0.249			***p*** **= 0.0125**
EGFP-Ala htau	untreated	CA3	B6	5	6	2.912±0.224	**F (3,20) = 4.276 ;** ***p*** **= 0.0174**	*p* = 0.8829	***p*** **= 0.0076**
EGFP-Ala htau	untreated	CA3	APP_SDL_	4	6	2.984±0.396			*p* = 0.0667
EGFP-PHP htau	untreated	CA1	B6	4	7	3.342±0.298	**F (3,24) = 5.250 ;** ***p*** **= 0.0063**	*p* = 0.6532	*p* = 0.1107
EGFP-PHP htau	untreated	CA1	APP_SDL_	4	6	3.155±0.429			*p* = 0.1091
EGFP-PHP htau	untreated	CA3	B6	5	6	3.578±0.450	F (3,20) = 2.724 ; *p* = 0.0714	*p* = 0.1941	*p* = 0.1722
EGFP-PHP htau	untreated	CA3	APP_SDL_	4	6	2.851±0.352			*p* = 0.0621
Construct	Condition	Region	Genotype	Mice (n)	Cells (n)	Mean ± s.e.m (based on cell number) in mm	One way ANOVA (together with EGFP untreated, both genotypes, same region)	*p* values (B6 vs. APP)	*p* values ( vs. untreated EGFP same region and genotype)
EGFP	CPP	CA1	B6	6	7	3.597±0.207	F (3,23) = 0.4707 ; *p* = 0.7056	*p* = 0.7702	*p* = 0.4144
EGFP	CPP	CA1	APP_SDL_	6	6	3.787±0.334			*p* = 0.5514
EGFP	CPP	CA3	B6	5	5	3.603±0.244	F (3,22) = 2.806 ; *p* = 0.0634	*p* = 0.7762	*p* = 0.0473
EGFP	CPP	CA3	APP_SDL_	5	6	3.474±0.382			*p* = 0.0974
EGFP	EpoD	CA1	B6	6	6	3.110±0.307	F (3,23) = 0.2951 ; *p* = 0.8285	*p* = 0.4532	*p* = 0.9960
EGFP	EpoD	CA1	APP_SDL_	7	7	3.591±0.168			*p* = 0.7353
EGFP	EpoD	CA3	B6	6	6	2.995±0.268	**F (3,23) = 5.693 ;** ***p*** **= 0.0046**	***p*** **= 0.0438**	***p*** **= 0.0006**
EGFP	EpoD	CA3	APP_SDL_	6	6	3.851±0.287			*p* = 0.3939
B. Number of branching points									
Construct	Condition	Region	Genotype	Mice (n)	Cells (n)	Mean ± s.e.m (based on cell number)	Student’s *t* test		
							*p* values (B6 vs. APP)		
EGFP	untreated	CA1	B6	5	9	31±4.11	*p* = 0.571		
EGFP	untreated	CA1	APP_SDL_	5	5	36±7.22			
EGFP	untreated	CA3	B6	7	8	39±3.83	*p* = 0.203		
EGFP	untreated	CA3	APP_SDL_	5	7	33±1.90			
EGFP	untreated	DG	B6	6	6	11±2.68	*p* = 0.261		
EGFP	untreated	DG	APP_SDL_	5	7	7±0.57			
EGFP-htau	untreated	CA1	B6	4	7	36±3.58	***p*** **= 0.016**		
EGFP-htau	untreated	CA1	APP_SDL_	6	8	23±3.05			
EGFP-htau	untreated	CA3	B6	5	6	40±3.24	*p* = 0.185		
EGFP-htau	untreated	CA3	APP_SDL_	5	6	34±2.24			
EGFP-htau	untreated	DG	B6	5	10	8±4.49	*p* = 0.933		
EGFP-htau	untreated	DG	APP_SDL_	7	17	8±2.73			
Construct	Condition	Region	Genotype	Mice (n)	Cells (n)	Mean ± s.e.m (based on cell number)	One way ANOVA (together with EGFP-htau untreated, both genotypes, same region)	*p* values (B6 vs. APP)	*p* values ( vs. untreated EGFP-htau same region and genotype)
EGFP-htau	DAPT	CA1	B6	4	6	36±2.11	**F (3,24) = 4.920 ;** ***p*** **= 0.0084**	*p* = 0.2443	*P* > 0.9999
EGFP-htau	DAPT	CA1	APP_SDL_	4	7	31±2.00			*p* = 0.0511
EGFP-htau	DAPT	CA3	B6	5	6	30±1.95	**F (3,22) = 8.519 ;** ***p*** **= 0.0006**	*p* = 0.1167	***p*** **= 0.0058**
EGFP-htau	DAPT	CA3	APP_SDL_	4	8	25±1.51			***p*** **= 0.0076**
EGFP-htau	CPP	CA1	B6	5	7	32±4.34	F (3,25) = 2.434 ; *p* = 0.0885	*p* = 0.4497	*p* = 0.4497
EGFP-htau	CPP	CA1	APP_SDL_	6	7	28±3.53			*p* = 0.3310
EGFP-htau	CPP	CA3	B6	6	6	32±2.77	**F (3,23) = 5.381 ;** ***p*** **= 0.0059**	*p* = 0.1041	*p* = 0.0509
EGFP-htau	CPP	CA3	APP_SDL_	7	9	26±2.19			***p*** **= 0.0339**
EGFP-htau	EpoD	CA1	B6	7	8	27±1.83	**F (3,27) = 4.824 ;** ***p*** **= 0.0081**	*p* = 0.5753	***p*** **= 0.0203**
EGFP-htau	EpoD	CA1	APP_SDL_	8	8	25±1.31			*p* = 0.5753
EGFP-htau	EpoD	CA3	B6	7	8	29±2.86	**F (3,23) = 4.362 ;** ***p*** **= 0.0143**	*p* = 0.4458	***p*** **= 0.0120**
EGFP-htau	EpoD	CA3	APP_SDL_	7	7	26±2.93			*p* = 0.0668
EGFP-Ala htau	untreated	CA1	B6	5	7	26±3.41	**F (3,24) = 3.655 ;** ***p*** **= 0.0266**	*p* = 0.1135	***p*** **= 0.0430**
EGFP-Ala htau	untreated	CA1	APP_SDL_	4	6	34±3.12			***p*** **= 0.0288**
EGFP-Ala htau	untreated	CA3	B6	5	6	25±3.06	**F (3,20) = 4.572 ;** ***p*** **= 0.0135**	*p* = 0.5032	***p*** **= 0.0028**
EGFP-Ala htau	untreated	CA3	APP_SDL_	4	6	28±3.72			*p* = 0.1878
EGFP-PHP htau	untreated	CA1	B6	4	7	32±3.28	**F (3,24) = 3.736 ;** ***p*** **= 0.0246**	*p* = 0.1469	*p* = 0.3815
EGFP-PHP htau	untreated	CA1	APP_SDL_	4	6	25±2.45			*p* = 0.6630
EGFP-PHP htau	untreated	CA3	B6	5	6	38±6.57	F (3,20) = 0.6744 ; *p* = 0.5778	*p* = 0.3253	*p* = 0.7762
EGFP-PHP htau	untreated	CA3	APP_SDL_	4	6	31±6.14			*p* = 0.6702
Construct	Condition	Region	Genotype	Mice (n)	Cells (n)	Mean ± s.e.m (based on cell number)	One way ANOVA (together with EGFP untreated, both genotypes, same region)	*p* values (B6 vs. APP)	*p* values ( vs. untreated EGFP same region and genotype)
EGFP	CPP	CA1	B6	6	7	34±3.15	F (3,23) = 0.3279 ; *p* = 0.8052	*p* = 0.7518	*p* = 0.6011
EGFP	CPP	CA1	APP_SDL_	6	6	36±2.74			*p* > 0.9999
EGFP	CPP	CA3	B6	5	5	33±3.24	F (3,22) = 2.872 ; *p* = 0.0594	*p* = 0.1730	*p* = 0.2132
EGFP	CPP	CA3	APP_SDL_	5	6	26±3.16			*p* = 0.1396
EGFP	EpoD	CA1	B6	6	6	25±2.16	F (3,23) = 1.069 ; *p* = 0.3814	*p* = 0.3033	*p* = 0.2778
EGFP	EpoD	CA1	APP_SDL_	7	7	31±0.57			*p* = 0.4130
EGFP	EpoD	CA3	B6	6	6	23±3.24	**F (3,23) = 4.970 ;** ***p*** **= 0.0084**	***p*** **= 0.0141**	***p*** **= 0.0010**
EGFP	EpoD	CA3	APP_SDL_	6	6	35±1.99			*p* = 0.6501

### The microtubule-stabilizing drug EpoD induces dendritic simplification in control cultures

**Fig. 5:**
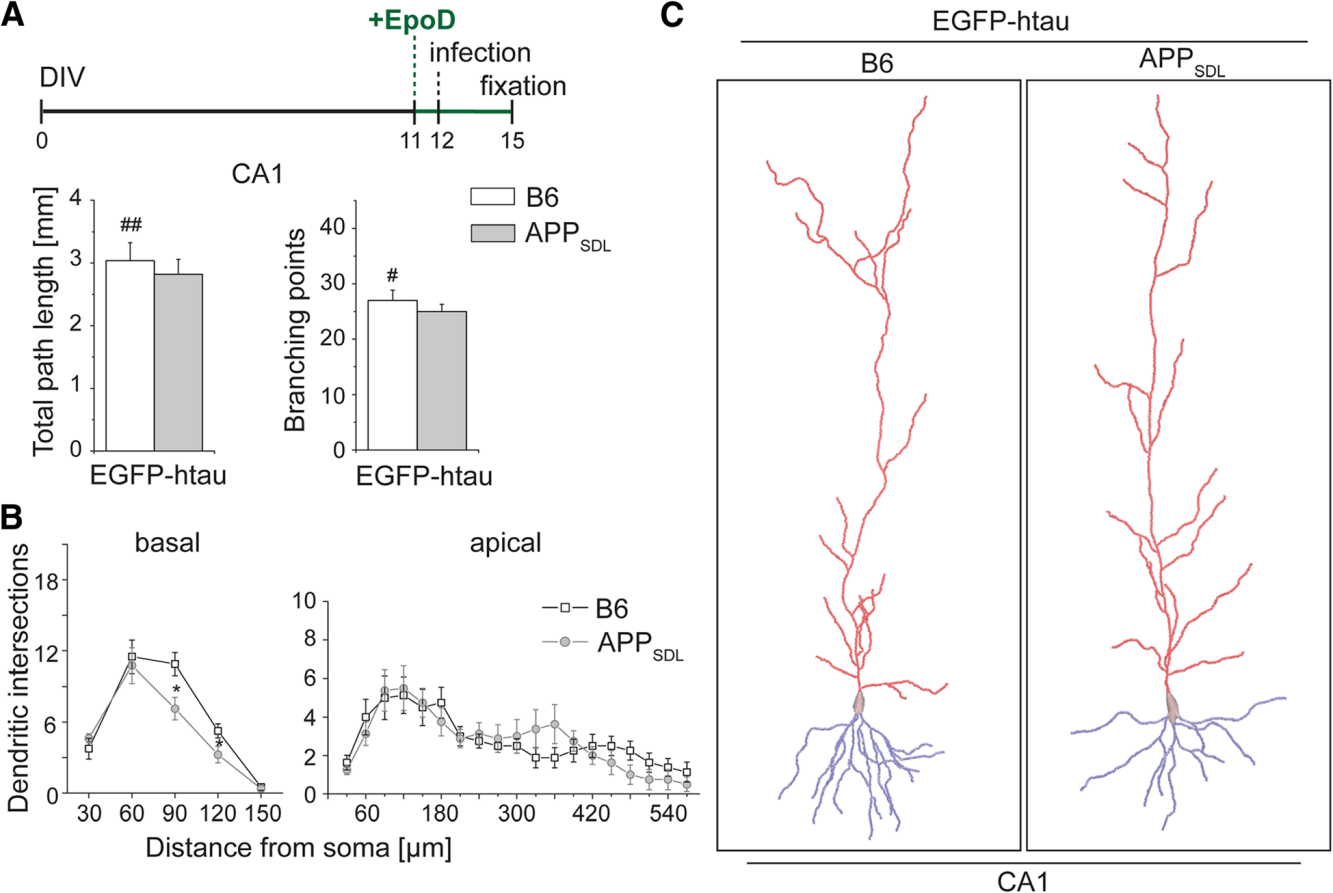
Effect of the microtubule-stabilizing drug EpoD on dendritic simplification. **a** Total path length and number of branching points of CA1 neurons in the presence of EpoD. Schematic representation of the time line of the experiment is shown on top. EpoD causes dendritic simplification on a non-transgenic background. **b**, **c** 3D reconstructions of representative CA1 neurons (**c**) and Sholl analysis (**b**) from APP_SDL_ transgenic and non-transgenic (B6) mice in the presence of EpoD illustrating similar complexity in the basal (blue) and apical (red) part of the dendritic tree. (#, ##) indicate significant decrease compared to the respective condition without EpoD. Statistical evaluation was performed using one way ANOVA with post hoc Fisher’s LSD test for multiple comparisons (**a**) and Student’s *t* test for comparison of the two genotypes (**b**). Values are shown as mean and s.e.m. *(#), *p* < 0.05, (##), *p* < 0.01. See Table 2 for details on the statistics

Data from this study could suggest that Aβ induces microtubule destabilization in a tau-dependent manner, which results in a decrease of dendritic complexity. If true, one would expect that microtubule-stabilizing drugs would prevent dendritic simplification. To test this hypothesis we treated the cultures prior to infection with the microtubule-stabilizing drug epothilone D (EpoD), a brain-penetrant small molecule potential therapeutic candidate for AD [28, 29]. Epothilones may have neuroprotective activity; however dose-dependent neurotoxic effects have also been reported [30]. In pilot experiments we observed loss of neurons in control cultures at concentrations of 1 and 5 nM of EpoD indicating some toxicity for the cells. The number of neurons was not affected at 0.2 nM EpoD, a concentration where still induction of microtubule polymerization was observed in model neurons (data not shown). Thus this concentration was subsequently used for the experiments. Surprisingly, EpoD induced dendritic simplification in control cultures as indicated by significantly decreased total path length and branching compared to the respective untreated cultures (Fig. 5a, Table 2). No obvious differences in the dendritic morphology between htau-expressing APP-transgenic and control cultures were observed, which was – except some simplification in basal dendrites – confirmed by Sholl analysis (Fig. 5b, c).

**Table 2:** Effect of drug treatment on dendritic tau levels. Dendritic tau levels in 2^nd^ or 3^rd^ order of CA1 neurons expressing the respective construct were determined as described in “Methods”. For every neuron, 3–4 measurements were performed and averaged. Statistical evaluation was performed using two-tailed, unpaired Student’s *t* test for untreated cultures, and one way ANOVA with post hoc Fisher’s LSD test for multiple comparisons of drug treatments or different tau constructs. Note that alpha levels of ANOVA did not reach significance (*p*≤0.05) for any condition

Construct	Condition	Region	Genotype	Mice (n)	Cells (n)	Mean ± s.e.m (based on cell number) of normalized intensity values	Student’s *t* test		
							*p* values (B6 vs. APP)		
EGFP-htau	untreated	CA1	B6	3	5	1.061±0.099	*p* = 0.6210		
EGFP-htau	untreated	CA1	APP_SDL_	5	5	1.111±0.120			
Construct	Condition	Region	Genotype	Mice (n)	Cells (n)	Mean ± s.e.m (based on cell number) of normalized intensity values	One way ANOVA (together with EGFP-htau untreated, both genotypes)	*p* values (B6 vs. APP)	*p* values (vs. untreated same genotype)
EGFP-htau	DAPT	CA1	B6	3	6	0.827±0.066	F (3,17) = 2.183 ; *p* = 0.1275	*p* = 0.0273	*p* = 0.2007
EGFP-htau	DAPT	CA1	APP_SDL_	4	5	1.197±0.154			*p* = 0.5981
EGFP-htau	EpoD	CA1	B6	4	5	1.119±0.138	F (3,16) = 0.2108 ; *p* = 0.8874	*p* = 0.8533	*p* = 0.5763
EGFP-htau	EpoD	CA1	APP_SDL_	5	5	1.148±0.066			*p* = 0.8135
EGFP-htau	CPP	CA1	B6	5	5	0.945±0.176	F (3,16) = 1.334 ; *p* = 0.2984	*p* = 0.3752	*p* = 0.6129
EGFP-htau	CPP	CA1	APP_SDL_	4	5	0.793±0.019			*p* = 0.0745
EGFP-Ala htau	untreated	CA1	B6	5	5	1.083±0.135	F (3,16) = 0.6240 ; *p* = 0.6098	*p* = 0.2952	*p* = 0.7390
EGFP-Ala htau	untreated	CA1	APP_SDL_	5	5	0.917±0.068			*p* = 0.2240
EGFP-PHP htau	untreated	CA1	B6	3	5	1.174±0.064	F (3,16) = 0.5229 ; *p* = 0.6727	*p* = 0.9881	*p* = 0.2937
EGFP-PHP htau	untreated	CA1	APP_SDL_	3	5	1.172±0.080			*p* = 0.6495

### Expression of phosphoblocking tau induces dendritic simplification *per se* and increases microtubule stability

**Fig. 6:**
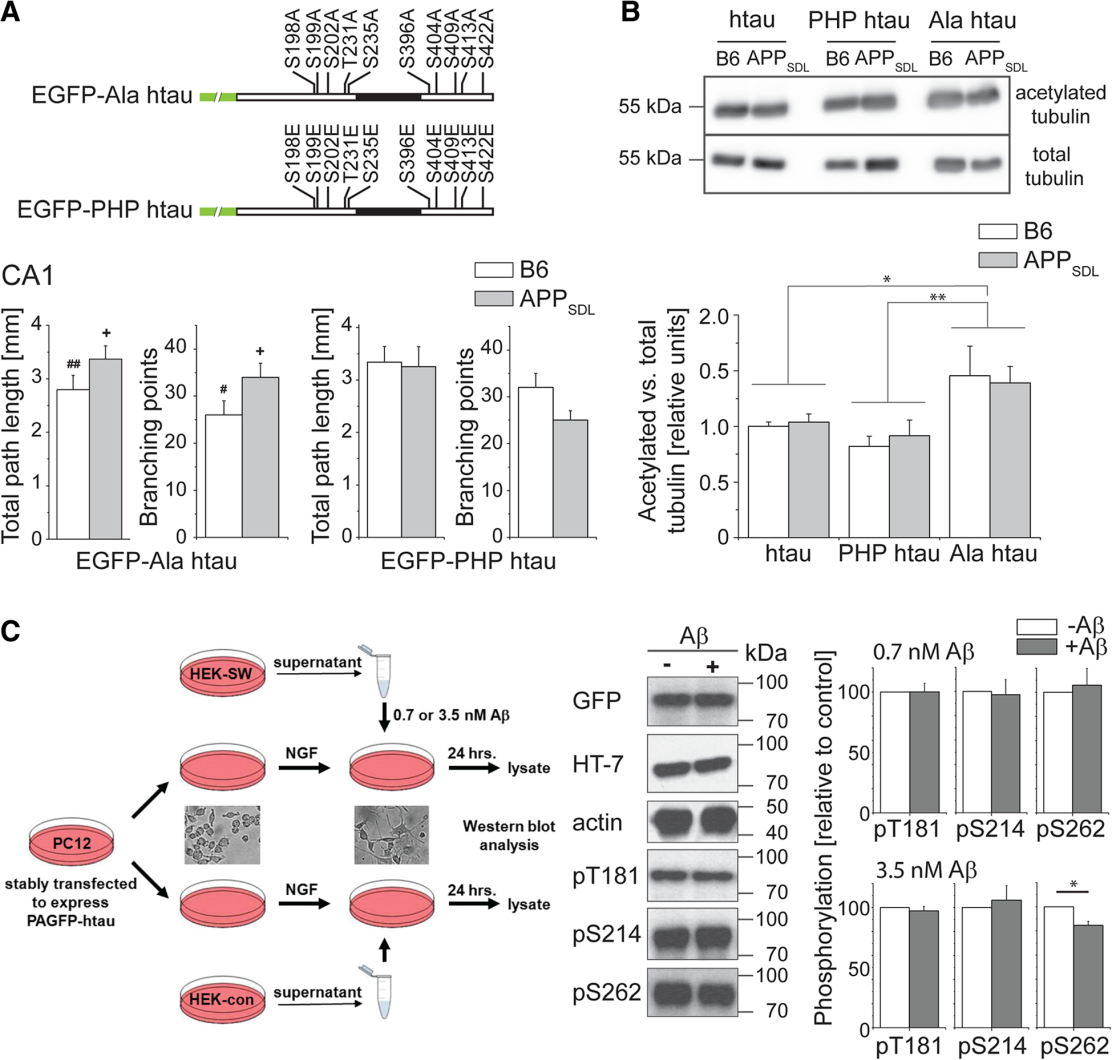
Effect of phosphoblocking and phosphomimicking tau on dendritic simplification. **a** Total path length and number of branching points of CA1 neurons that express phosphoblocking (Ala htau) or phosphomimicking tau (PHP htau). Schematic representations of the respective construct are shown on the top. Mutated residues are indicated. Ala htau causes dendritic simplification on a non-transgenic background, whereas PHP htau behaves neutral. **b** Ratio of acetylated to total tubulin as a marker for microtubule stabilization. Immunoblots showing staining against acetylated tubulin and total tubulin (*top*) and the respective quantitation (*bottom*). Expression of Ala htau causes stabilization of microtubules. **c** Effect of low nanomolar concentrations of naturally secreted Aβ on the phosphorylation of tau in model neurons. A schematic representation of the experimental approach is shown to the left. Neuronally differentiated PC12 cells expressing PAGFP-tagged human tau are incubated with a supernatant from Aβ-expressing cells (HEK-SW) or the respective controls (HEK-con). Representative Western blots and quantification of phosphorylation at selected sites is shown to the right. 3.5 nM Aβ induce dephosphorylation of tau at S262. (+) indicate significant increase, (#, ##) significant reduction compared to neurons expressing wildtype human tau. Statistical evaluation was performed using one way ANOVA with post hoc Fisher’s LSD test for multiple comparisons (**a**) and Student’s *t* test for comparison of the constructs (**b**) and the two genotypes (**c**). Values are shown as mean and s.e.m. See Table 1 for details on the statistics. For (**c**), 3–10 Western blot experiments were evaluated per condition. *(#)(+), *p* < 0.05, **(##), *p* < 0.01

Since microtubule stabilization did not have any effect in Aβ-producing cultures, we explored whether tau phosphorylation, which is known to affect microtubule dynamics, is at all involved in mediating dendritic simplification. We employed a phosphoblocking (Ala htau) and a phosphomimicking (PHP htau) construct in which 10 AD-relevant sites were modified to alanine to prevent, or to glutamate to simulate, phosphorylation at these residues (Fig. 6a, top) [31]. Surprisingly, expression of Ala htau induced dendritic simplification *per se* whereas the phosphomimicking PHP htau did not induce morphological changes (Fig. 6a, bottom, Table 1). This is in sharp contrast to the previous observation that PHP tau is the active species to induce cell death, and importantly indicates that the development of dendritic simplification and tau-dependent cell death are mechanistically distinct. The finding that the effect of Ala htau expression closely resembles the impact of EpoD-treatment could suggest that Ala htau induces dendritic simplification by hyperstabilizing dendritic microtubules. To test this hypothesis, we determined the influence of wt htau, Ala htau and PHP htau expression on microtubule stability by determining the ratio of acetylated to total tubulin, since tubulin acetylation is considered to be a marker for microtubule stability [32]. Expression of Ala htau induced a significantly increased ratio of acetylated to total tubulin compared to wt htau or PHP htau expressing cultures (Fig. 6b). This indicates that non-phosphorylatable htau induces dendritic simplification due to the inherent activity to promote microtubule stabilization. It should however be noted that Ala htau expression prevented dendritic simplification in CA1 neurons from APP_SDL_ transgenic animals (Fig. 6a, bottom), which might indicate that it counteracts Aβ-induced microtubule destabilization in these neurons.

Generally, higher Aβ concentrations are considered to increase rather than decrease tau phosphorylation, but the effect of low concentrations of secreted Aβ on the phosphorylation of human tau is less clear. A previous study using a coculture system has shown that secreted Aβ induces an increased phosphorylation at the AT8 (S202/T205) and the AT180 epitope (T231/S235) [33]. The concentration of Aβ was comparable with that found in cerebrospinal fluid of AD patients, probably in the higher nanomolar range. However, a rough estimate suggests that the amount of Aβ in the brain of APP_SDL_ transgenic mice that have been used for preparation of the organotypic slices is in the low nanomolar range (L. Bakota, unpublished observation), which reflects more a presymptomatic scenario. To test the hypothesis that low nanomolar concentrations of naturally secreted Aβ cause a change in the phosphorylation pattern of tau that might lead to increased microtubule stabilization, we turned to a cellular model, where PAGFP-tagged human tau is stably expressed in rat PC12 cells (Fig. 6c). The cells were seeded in a defined density, neuronally differentiated with NGF and incubated with a supernatant of APP_Swe_-transfected HEK cells. Western blot analysis showed that 3.5 nM Aβ caused a decreased phosphorylation at S262, while some other sites such as T181 and S214 were not affected. Interestingly, S262 is located in the microtubule-binding region of tau and phosphorylation of S262 largely abolishes its interaction with microtubules [34]. Thus, even a small decrease in S262 phosphorylation could induce tau-dependent microtubule stabilization thereby promoting dendritic simplification.

## Discussion

The major findings of our current study comprise: (1) dendritic simplification is induced by Aβ and mediated by tau through NMDAR activation in a spatially highly restricted manner; (2) dendritic simplification is mechanistically distinct from other neurodegenerative events; and (3) dendritic simplification involves microtubule stabilization by dendritic tau, which becomes dephosphorylated at certain sites.

In contrast to spine changes and neuron loss, the mechanisms that contribute to dendritic simplification are much less studied. The pattern of dendritic arborization crucially determines the final integration of inputs and information processing. Thus, it is clear that a reduction of total dendritic path length by about 40 % as we observed it in principal neurons of the CA1 region (see Fig. 2a), has a major impact on synaptic connectivity even if the density of spines would remain unchanged.

It is well established that neuronal activity and activation of NMDARs play a key role in the regulation of dendritic arbor elaboration and dynamics as NMDAR-dependent neurotransmission induces an increased complexity of dendritic branches [35] and hypofunctional NMDARs lead to dendritic simplification [36]. This is in agreement with our observation that Aβ induces dendritic simplification through NMDARs and that morphological changes occur at sites of Schaffer collateral input, i.e. at glutamatergic synapses. Downstream signaling pathways may involve modulation of dendritic Ca^2+^-dependent plasticity and may finally be mediated through regulation of cytoskeletal dynamics. Previous studies have shown that members of the Rho family of small GTPases such as RhoA and Cdc42 influence dendritic development, possibly by functionally linking surface receptor activation to the organization of the actin cytoskeleton [37]. Very recently it also has been shown that a RhoA signaling pathway regulates the formation of Golgi outposts that contribute to dendritic growth and branch dynamics [38]. Interestingly, the activity level of RhoA appears also to modify the phosphorylation state of tau [39]. On the other side, tau belongs to the class of intrinsically disordered proteins, which are known to serve as hubs in cellular protein-protein interaction networks and are involved in signaling [40]. Thus, it is conceivable that tau phosphorylation influences various signal transduction mechanisms that are involved in the complex process of dendritic arborization.

**Fig. 7:**
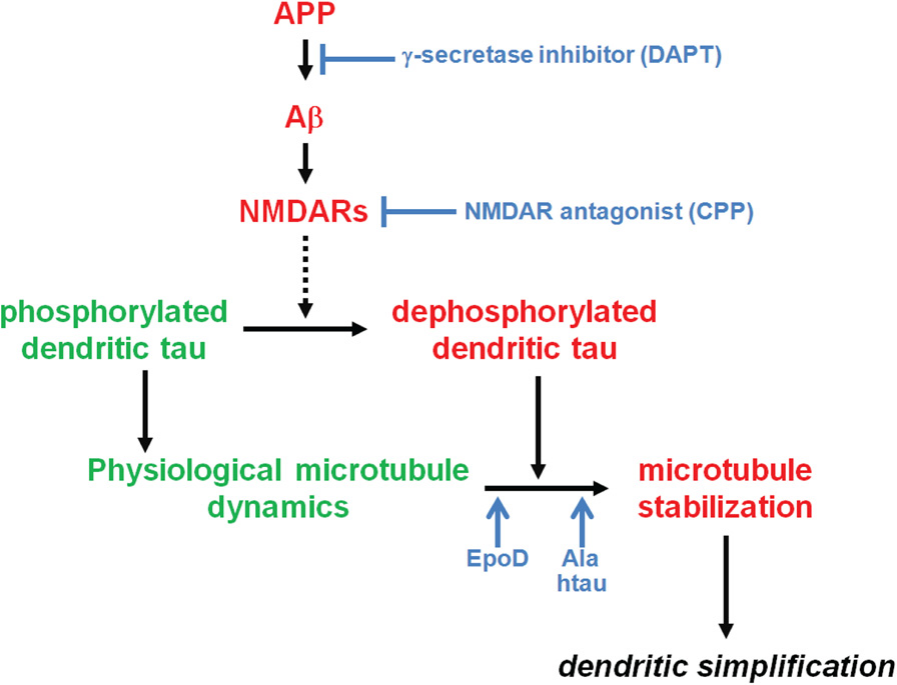
Schematic representation showing the proposed pathway that mediates dendritic simplification. The formation of Aβ and NMDAR activity are required for dendritic simplification since blocking of Aβ production or inhibition of NMDAR activity abolish the pathology. NMDAR activation leads to pathologic dephosphorylation of dendritic tau, which shows at physiological conditions a higher phosphorylation state. Dephoshorylation increases tau’s activity to stabilize dendritic microtubules, an activity that is mimicked by the microtubule-stabilizing drug EpoD or phosphoblocking tau (Ala htau). Continuous lines show direct effects, and dashed lines show indirect effects with potential intermediate steps

We have provided evidence that Aβ induces changes in htau by acting on NMDARs, which then lead to local modulation of microtubule dynamics (Fig. 7). This provokes the question, how the mechanisms of dendritic simplification relate to the other facets of the neurodegenerative triad that occur during AD, *i.e.*, to spine changes and neuron loss. We and others have previously reported that soluble Aβ causes loss of spines in an NMDAR-dependent manner, but independent of tau [5, 26]. This contrasts with the requirement of tau for the induction of dendritic simplification and indicates that different mechanisms operate to mediate spine alterations on the one hand, and changes of dendritic arborization on the other. Neuron loss requires a combined action of Aβ and tau [5, 41, 42], indicating that dendritic simplification and loss of neurons share the engagement of tau. However, tau-dependent cell loss occurred in the CA3 region, but not in CA1 neurons [21], whereas dendritic simplification was mostly evident in CA1 and not in CA3. The inverse regional relationship argues against the hypothesis that dendritic simplification is a forerunner to neuronal loss [9]. Furthermore, neuron loss was blocked in the presence of phosphoblocking tau [5], whereas the same construct induced dendritic simplification *per se* as shown in the present study. Thus the data indicate that dendritic simplification is mechanistically distinct from the other two features of the neurodegenerative triad.

Importantly, we demonstrated that dendritic simplification was induced in a highly localized manner in the hippocampus. In AD patients, major dendritic regression of CA1 neurons was found [11], whereas no reduction of dendritic extent in CA3 pyramidal neurons occurred [12, 13]. Remarkably, the region-specificity was recapitulated in our model suggesting that this model provides a useful system to analyze the mechanism of region-specific dendritic simplification. Since soluble Aβ is globally present, dendritic tau appears to play a decisive role in mediating region specific effects, which might differ between cells and subcellular microdomains. It has previously been shown that tau is present to a major extent in dendrites where it generally has a higher phosphorylation state than in the axon [19, 43]. In this study we have demonstrated that a phosphoblocking tau construct leads to increased microtubule stabilization that causes dendritic simplification suggesting that conditions, which decrease the phosphorylation state of dendritic tau at certain sites can be harmful. None of the tau constructs or treatment conditions changed the dendritic tau level suggesting that a redistribution of tau does not play a role in mediating dendritic simplification (Table 2). Using a cellular model system we showed in this study that low nanomolar concentrations of secreted Aβ cause a decreased phosphorylation at S262, a site that, when phosphorylated, abolishes tau’s interaction with microtubules [34].

Interestingly, phosphorylated S262 (but not T181 or S214) is a preferential target for the major brain phosphatase calcineurin (PP2B) [44, 45], which has previously been shown to be activated by Aβ in a NMDAR-dependent pathway [5, 26] and was reported to be involved in dendritic simplification [8]. Our data could suggest that phosphorylation at S262 might be a target for a selective intervention in the mechanisms that lead to dendritic simplification. Furthermore we have shown that NMDAR activation is involved in inducing dendritic simplification and that dendritic simplification takes place at sites of Schaffer collateral inputs where postsynaptic LTP occurs [46].

## Conclusions

Using an ex vivo model we showed that (1) dendritic simplification is induced by Aβ and mediated by tau through NMDAR activation in a spatially highly restricted manner; (2) dendritic simplification is mechanistically distinct from other neurodegenerative events; and (3) dendritic simplification involves microtubule stabilization by dendritic tau, which is dephosphorylated at specific sites. The proposed pathway that mediates dendritic simplification is shown in Fig. 7.

The finding that the mechanisms inducing and mediating dendritic simplification are distinct from other AD related abnormalities could be relevant for the development of treatments to slow down the neurodegenerative triad and should be taken into account when testing new approaches. In particular, treatments leading to an overall decrease of tau phosphorylation [47] might have a negative impact on neuronal connectivity since they could induce dendritic simplification.

## Methods

### Animals

Heterozygous APP_SDL_ transgenic mice [48] expressing human APP_695_ with three familial AD mutations were used. APP_SDL_ mice express equimolar concentrations of Aβ40 and Aβ42 already at embryonal age [49]. Genotyping was performed as described previously [5]. APP_SDL_ transgenic and C57BL/6 J (B6) control animals were maintained and sacrificed according to National Institutes of Health Guidelines and German Animal Care Regulations.

### Materials

Chemicals were purchased from Sigma (Steinheim, Germany), culture medium and supplements from Sigma and Invitrogen (Darmstadt, Germany), culture dishes and plates from Nunc (Langenselbold, Germany), and membrane culture inserts from Millipore (Billerica, MA, USA). γ-secretase inhibitor *N-*[N-(3,5-difluorophenacetyl)-1-alanyl]-*S*-phenylglycine *t*-butyl ester (DAPT) was purchased from Merck (Darmstadt, Germany). Epothilone D (EpoD) was prepared as previously described [50, 51]. The spectroscopic properties of the compound were identical to those reported in the literature. Compound purity was >95 % as determined by LC-MS and NMR analyses.

### Sindbis virus constructs

Construction of virus was performed as described previously [21]. The following constructs were used for the experiments: pSinRep5-EGFP, pSinRep5-EGFP-352wt htau, pSinRep5-EGFP-352 Ala htau, pSinRep5-EGFP-352 PHP htau. For Ala and PHP htau, respectively, 10 sites (Ser198, Ser199, Ser202, Thr231, Ser235, Ser396, Ser404, Ser409, Ser413, and Ser422) were mutated to alanine to block or to glutamate to mimic phosphorylation at the respective residues [31].

### Organotypic hippocampal slice cultures and Sindbis virus infection

Hippocampal slice cultures were prepared from mice of either sex and processed as described previously [5, 52]. On day 12 in vitro slice cultures were infected with Sindbis virus. For some experiments, slices were treated with 0.5 μM DAPT, 20 μM 3-(2-carboxypiperazin-4-yl)propyl-1-phosphonic acid (CPP), or 0.2 nM EpoD starting on day 11 in vitro. Cultures were fixed at day 3 post infection. For preparation of lysates, cultured hippocampal slices were visually inspected on day 15 *in vitro*. Slices with similar infection rates were lysed in RIPA buffer as described previously [5].

### Confocal microscopy, image processing and semi-automated analysis of neuronal morphology

Confocal high-resolution microscopy of complete principal hippocampal neurons was performed as described previously [22]. Each single neuron was imaged in 8 to 12 overlapping image stacks with voxel size of 0.14 × 0.14 × 0.44 μm in *x-y-z* directions. 3D reconstruction of whole neurons was performed using Neuromantic software (University of Reading, Reading, UK) in semi-automated mode.

For the determination of dendritic tau levels, non overlapping basal dendritic segments of 2nd or 3rd order of CA1 neurons expressing EGFP-tagged tau constructs were selected. Image stacks between first and last appearance of the respective dendritic segment were projected onto a single layer and mean intensity values in the middle of the process were determined and corrected for the detector gain.

### Culture of PC12 and HEK cells

PC12 cells stably transfected to express PAGFP-tagged wt 352 htau were cultured in serum-DMEM in the presence of 250 μg/ml Geneticin as described previously [53]. Cells were plated onto 10-cm collagen-coated TC-plates at 2 × 10^4^ cells/cm^2^. On the next day, medium was replaced with low serum DMEM (DMEM with 1 % (vol/vol) serum) containing 100 ng/ml 7S mouse NGF (Alomone Laboratories). After 4 days, medium was replaced with serum-free medium (NB/B-27, Thermo Fisher Scientific) containing 100 ng/ml 7S mouse NGF and supernatant of Aβ-producing HEK cells (HEK-SW) to yield 0.7 or 3.5 nM of Aβ. Same amounts of supernatant of untransfected HEK cells (HEK-con) were added as control. Cell lysates were prepared 24 h. later as described by [53].

HEK-SW cells (HEK 293-APP_swe_; [54]), which were stably transfected with APP carrying the Swedish familial AD mutation resulting in increased Aβ secretion, were obtained from C. Haass (University of Munich) and cultured in DMEM containing 10 % fetal calf serum. As a control, HEK 293FT cells (Life Technologies) were used. Cells were cultured until 60–70 % confluency, medium was exchanged and the supernatant collected 24 h. later. The supernatant was filtered (0.22 μm pore size) and shock frozen in aliquots in liquid nitrogen. The concentration of Aβ_1–40_ and Aβ_1–42_ was determined by ELISAs according to the manufacturer’s description (EZHS40, EZHS42; Millipore). The ratio of Aβ_1–42_ to Aβ_1–40_ in the supernatant of HEK-SW cells was 1:4.

### Immunoblot analysis

Same volumes of lysates were subjected to SDS-PAGE, transferred to Immobilon-P (Millipore) and stained. Immunodetection used the following antibodies: Phosphorylation-independent tau antibodies: HT7 (human tau, mouse; Pierce); phosphorylation-dependent tau antibodies: AT270 (pThr181, mouse; Pierce), p214 (Ser-214, rabbit; Biosource), pS262 (pSer262, rabbit; Biosource). Also used were anti-GFP (rabbit; Invitrogen), anti-tubulin (mouse, DM1A), anti-acetylated tubulin (mouse; 6-11B-1), and anti-actin (mouse, JLA20; Amersham) antibodies. As secondary antibodies, peroxidase–conjugated goat anti–mouse and anti–rabbit antibodies (Jackson ImmunoResearch Laboratories, Inc.) were used. Detection using enhanced chemiluminiscence and quantitation were performed as described previously [17].

### Statistical analysis

Statistical evaluation was performed using one way analysis of variance (ANOVA) with post hoc Fisher's least significant difference (LSD) test for multiple comparisons and two-tailed, unpaired Student’s *t* test for comparison of the two genotypes at control conditions. For the tau phosphorylation experiments, one-sample Student’s *t* test was performed. Data are shown as mean and s.e.m. Sholl analysis [23] was performed separately for basal and apical parts of dendritic trees.

## Abbreviations

Aβ: Amyloid beta
ΑΝΟVΑ: Analysis of variance
ΑPP: Amyloid precursor protein
AD: Alzheimer’s disease
DIV: Days *in vitro*
EGFP: Enhanced green fluorescent protein
EpoD: Epothilone D
LSD: Least significant difference
NMDAR: NMDA receptor
